# Simultaneous Inhibition of Thrombosis and Inflammation Is Beneficial in Treating Acute Myocardial Infarction

**DOI:** 10.3390/ijms24087333

**Published:** 2023-04-15

**Authors:** Ian Vargas, Ryan P. Grabau, Junjie Chen, Carla Weinheimer, Attila Kovacs, William Dominguez-Viqueira, Adam Mitchell, Samuel A. Wickline, Hua Pan

**Affiliations:** 1 University of South Florida Heart Institute, University of South Florida, Tampa, FL 33602, USA; 2 Consortium for Translational Research in Advanced Imaging and Nanomedicine, Washington University School of Medicine, St. Louis, MO 63110, USA; 3 Cardiovascular Division, Washington University School of Medicine, St. Louis, MO 63110, USA; 4 Small Animal Imaging Laboratory, Moffitt Cancer Center, Tampa, FL 33612, USA; 5 Division of Rheumatology, Washington University School of Medicine, St. Louis, MO 63110, USA; 6 Department of Pathology and Immunology, Washington University School of Medicine, St. Louis, MO 63110, USA; 7 Department of Biomedical Engineering, Washington University in St. Louis, St. Louis, MO 63105, USA

**Keywords:** acute myocardial infarction, thrombosis, inflammation, inflammasome, PPACK, perfluorocarbon nanoparticles, fluorine magnetic resonance spectroscopy, fluorine magnetic resonance imaging

## Abstract

Myocardial ischemia reperfusion injury (IRI) in acute coronary syndromes is a condition in which ischemic/hypoxic injury to cells subtended by the occluded vessel continues despite successful resolution of the thrombotic obstruction. For decades, most efforts to attenuate IRI have focused on interdicting singular molecular targets or pathways, but none have successfully transitioned to clinical use. In this work, we investigate a nanoparticle-based therapeutic strategy for profound but local thrombin inhibition that may simultaneously mitigate both thrombosis and inflammatory signaling pathways to limit myocardial IRI. Perfluorocarbon nanoparticles (PFC NP) were covalently coupled with an irreversible thrombin inhibitor, PPACK (Phe[D]-Pro-Arg-Chloromethylketone), and delivered intravenously to animals in a single dose prior to ischemia reperfusion injury. Fluorescent microscopy of tissue sections and ^19^F magnetic resonance images of whole hearts ex vivo demonstrated abundant delivery of PFC NP to the area at risk. Echocardiography at 24 h after reperfusion demonstrated preserved ventricular structure and improved function. Treatment reduced thrombin deposition, suppressed endothelial activation, inhibited inflammasome signaling pathways, and limited microvascular injury and vascular pruning in infarct border zones. Accordingly, thrombin inhibition with an extraordinarily potent but locally acting agent suggested a critical role for thrombin and a promising therapeutic strategy in cardiac IRI.

## 1. Introduction

The development of therapies to limit cardiac ischemia reperfusion injury (IRI) in acute coronary syndromes represents a substantial unmet medical need [1,2,3]. Although early interventions that restore blood flow to the ischemic myocardium salvage the muscle at risk, the potential risk exists for up to ~50% additional loss of viable myocardium due to reperfusion injury that can culminate in accelerated cardiac remodeling, progressive heart failure, and death [4,5]. However, despite exhaustive research, clinical trials have failed to identify a single specific therapeutic intervention demonstrating definitive salvage of myocardium jeopardized by reperfusion injury [6,7].

A prevailing theory regarding post-myocardial infarction wound healing is that prolonged inflammation creates progressive “collateral damage” to viable but salvageable myocytes in infarct border zones that ultimately succumb to necrotic or apoptotic cell death [8,9]. Unfortunately, the pathophysiology of cardiac IRI remains incompletely understood, the phenomenon is complex [10], and the task of identifying the specific constellation(s) of molecular factors to target is daunting. In this light, a recent viewpoint publication by Davidson representing the EU COST CARDIOPROTECTION Action group suggests the opportunity for a shift in focus from traditional monotherapeutic approaches to more comprehensive multi-factorial therapies to attenuate the deleterious consequences of myocardial IRI [11]. Additionally, expanding the therapeutic purview to nonmyocyte cardiac matrix targets such as cardiac vasculature is receiving growing attention to attenuate vascular compromise and optimize wound healing [12,13,14,15,16,17]. Although the current standard-of-care for acute myocardial infarction includes combinations of potentially vascular protective agents to modulate thrombosis (e.g., heparin), platelet activation (e.g., aspirin, selective antiplatelet agents), and inflammation (e.g., aspirin), these regimens have not mitigated cardiac ischemia reperfusion injury [18]. Even direct thrombin inhibitors such as dabigatran do not appear to benefit IRI in experimental studies [19]. Moreover, the role of inflammatory and prothrombotic drivers in endothelial damage and vascular compromise remains relatively unexplored [20].

We suggest a novel explanation for the lack of efficacy of such cocktails against ischemia reperfusion injury: even in combination, these agents do not exert sufficiently rapid, potent, or long-lived local antithrombotic and antiinflammatory effects to prevent extensive microvascular damage in potentially viable but injured myocardial territories. We contend that the aggressive local activation of thrombin and platelets in areas of ischemic cardiac and vascular damage incites prothrombotic and inflammatory pathways that resist suppression with conventional agents when administered systemically in approved dosages. Indeed, thrombin itself may play the key role as a pleiotropic molecule that promotes clotting, platelet activation, inflammation, and vascular permeability, all contributing to cardiac ischemia reperfusion injury.

To test the corollary hypothesis that more powerful and persistent thrombin inhibition might improve cardiac ischemia reperfusion injury, we have designed long-acting antithrombin nanoparticles that are extraordinarily potent as compared with approved antithrombin agents, yet safer because they do not produce a sustained systemic anticoagulant effect or a bleeding diathesis [21]. We have previously shown that perfluorocarbon nanoparticles (PFC NP: ~200 nm) covalently coupled with ~13,650 individual PPACK (Phe[D]-Pro-Arg-Chloromethylketone) molecules protect the murine kidney from ischemia reperfusion injury and preserves vascular structures when given either as a single i.v. dose at the time of reperfusion [22] or a single dose after the onset of acute kidney injury [23]. In the present work, we extend this treatment regimen to cardiac ischemia reperfusion injury in mice and rats undergoing coronary artery occlusion for 90 min and 45 min, respectively. Cardiac function and geometry were improved, diverse inflammatory pathways were suppressed, and microvascular architecture was preserved at 24 h after ischemia reperfusion injury. These observations indicate that: (1) thrombin is a key promoter of inflammation and vascular damage in ischemia reperfusion injury, and (2) exceptionally potent antithrombin agents can effectively ameliorate the untoward consequences of reperfusion injury.

## 2. Results

### 2.1. Delivery of Perfluorocarbon Nanoparticles to the Heart with Acute Myocardial Infarction

**Figure 1:**
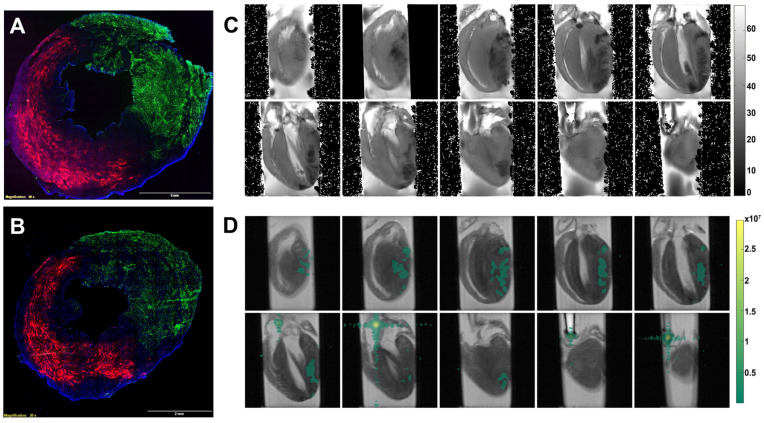
Delivery of perfluorocarbon (PFC) nanoparticles to the area at risk of the heart with ischemia reperfusion injury. (**A**,**B**) Representative microscopic fluorescent image demonstrates retention of PFC nanoparticles (Red) in the area-at-risk versus none in the normally perfused region (green) as delineated by lectin staining of blood vessels. Representative microscopic fluorescent image from mice 1 h (**A**) and 3 h (**B**) after reperfusion. DAPI was used for nuclei staining (blue). The size of the scale bar was 2 mm, and the magnification was 20×. (**C**) T2-weight MRI reveals edema in the area at risk. (**D**) ^19^F MRI (pseudo-colored green) illustrating the colocation of intact PFC nanoparticles with CE-core in regions with ischemia reperfusion injury.

Perfluorocarbon (PFC) nanoparticles serve as a multifunctional delivery platform comprising a hydrophobic perfluorocarbon core surrounded by a lipid surfactant monolayer [24,25]. The perfluorocarbon core also serves as a contrast agent for fluorine (^19^F) magnetic resonance imaging (MRI) and ^19^F magnetic resonance spectroscopy (MRS), which enables the detection and quantification of delivery of the perfluorocarbon nanoparticles [26,27,28]. Two different perfluorocarbons with distinct MRS signatures were used in this work, perfluorooctylbromide (PFOB) and perfluoro-15-crown-5 ether (CE), which have differential spectral fluorine resonances [29]. The MRS of CE has a single peak, which makes it a good candidate for MRS quantification and MRI. The lipid monolayer was functionalized with fluorescent molecules, Rhodamine, for microscopic localization of nanoparticle delivery to the heart. The Rhodamine-labeled PFOB nanoparticles were intravenously injected 15 min before the induction of ischemia by left anterior descending (LAD) coronary artery snare occlusion for 90 min in mice. The mouse hearts were excised either 1 (Figure 1A) or 3 (Figure 1B) hours after reperfusion and flushed with saline to remove the blood and any circulating nanoparticles. Then the LAD was reoccluded, and the hearts were perfused with Lycopersicon Esculentum lectin with DyLight 488 (green) to delineate vasculature in the normally perfused cardiac region versus the area at risk of the ischemia reperfusion injury. As illustrated in Figure 1A,B, retained PFOB NP (red) were observed throughout the area-at-risk after ischemia reperfusion injury, but none were present in normally perfused (green) territories.

To confirm the deposition of the PFC NP in a different species, a rat ischemia reperfusion injury model was used for MRI-based ^19^F imaging of PFC NP. Seven rats received PFC nanoparticles with CE-cores 15 min before ischemia induction. Two hours post-reperfusion, the rats were euthanized, and the hearts were perfused with saline before ^1^H and ^19^F MRI. Figure 1C shows the T2* maps of a heart that delineates areas of edema, where darker regions in the hearts indicate damage from ischemia reperfusion injury. Figure 1D is a ^19^F MRI (density image) with pseudo color displaying the CE signal superimposed on the corresponding anatomical T2* images with the heart, which is colocalized with the injured regions. These results confirmed the local delivery and persistence of intact PFC NP in the area at risk after i.v. administration.

### 2.2. PPACK PFC NP Treatment Preserved Ventricular Structure and Function

**Figure 2:**
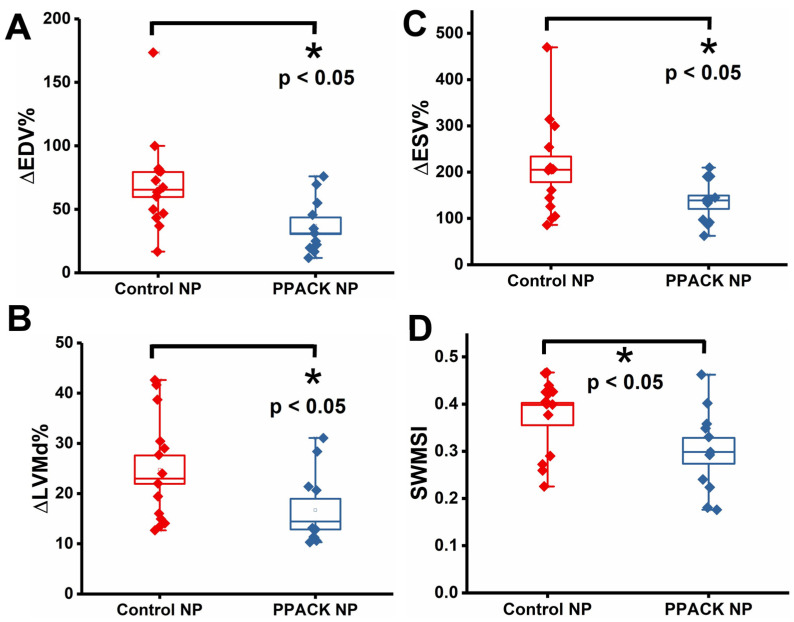
PPACK PFC NP treatment preserved ventricular structure and function. (**A**) Percentage change of End-Diastolic Volumes (∆EDV%). (**B**) Percentage change of End-Systolic Volumes (∆ESV%). (**C**) Percentage change of Left ventricular mechanical dyssynchrony (∆LVMD%). (**D**) segmental wall motion score index (SWMSI). (*n* = 11 PPACK NP and *n* = 14 control NP. Mean ± SEM, *: *p* < 0.05).

To evaluate PPACK PFC NP (PPACK NP) treatment versus plain PFC NP (control) (Control NP) for preserving cardiac function, 25 mice underwent 90 min LAD occlusion and reperfusion. Echocardiography 3–5 days before and 24 h after ischemia-reperfusion-injury was performed to measure the percentage change of (1) End-Diastolic Volumes (∆EDV%), (2) End-Systolic Volumes (∆ESV%), and (3) Left ventricular mechanical dyssynchrony (∆LVMD%). For ∆EDV%: 69.54% ± 9.86% in control NP vs. 37.04% ± 6.56% in PPACK NP treated mice (*p* < 0.05) (Figure 2A). For ∆ESV%: 206.29% ± 27.80% in control NP vs. 135.14% ± 14.41% in PPACK NP treated mice (*p* < 0.05) (Figure 2B). For ∆LVMD%: 24.79% ± 2.82% in control NP vs. 16.72% ± 2.26% in PPACK NP treated mice (*p* < 0.05) (Figure 2C). These results suggested that the PPACK NP treatment attenuated cardiac remodeling and improved functional metrics as early as 24 h after ischemia compared to mice receiving control nanoparticles.

A segmental wall motion score index (SWMSI) was calculated from echocardiographic images. SWMSI in control NP and PPACK NP groups were 0.39 ± 0.02 and 0.30 ± 0.03, respectively, with *p* < 0.05 (Figure 2D). As SWMSI is known to correlate closely with infarct size measured by histology [30,31,32], 24 h after ischemia reperfusion injury, the PPACK NP-treated group exhibited significantly smaller infarct compared with the control NP group.

### 2.3. PPACK Perfluorocarbon Nanoparticles Treatment Reduced Thrombin Deposition in the Border Zone Areas

**Figure 3:**
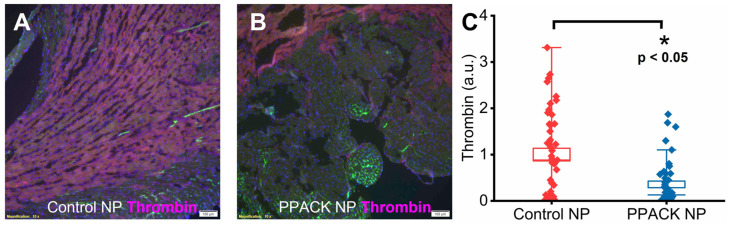
PPACK PFC NP treatment reduced thrombin deposition in border zones. Thrombin staining (red) in the heart from control NP-treated (**A**) and PPACK NP-treated (**B**) mice. Green fluorescence signal indicates blood vessels in the normal region of the heat. DAPI was used for nuclei staining (blue). The size of the scale bar was 100 µm, and the magnification was 10×. (**C**) Thrombin content in border zone regions of interest for the control NP or PPACK NP treatment. (*n* = 3 mice per group *n* = 45 PPACK NP and *n* = 45 control NP regions of interest. Mean ± SEM; nonparametric analysis on *n* = 3; *: *p* < 0.05).

We have reported previously that PPACK PFC NP reduces the deposition of thrombin in kidneys subject to ischemic injury [22]. In border zone territories, randomly selected regions of interest (*n* = 15 regions per heart in midwall ventricular slices exhibiting discrete normal and injured segments) were analyzed for thrombin content 24 h after reperfusion injury. Significantly greater thrombin deposition was observed for the control NP treatment (Figure 3A) than for the PPACK NP treatment (Figure 3B). For semi-quantification, the intensity of the thrombin signal in the border zone regions from microscopic images of PPACK NP-treated mice was normalized to that of control NP-treated mice. The normalized thrombin signal was 1 ± 0.14 and 0.36 ± 0.07 in the border zone of hearts from the control NP and PPACK NP-treated mice, respectively, indicating a reduction in thrombin deposition of 64% (*p* < 0.05).

### 2.4. PPACK Perfluorocarbon Nanoparticles Treatment Preserved Border Zone Vasculature

**Figure 4:**
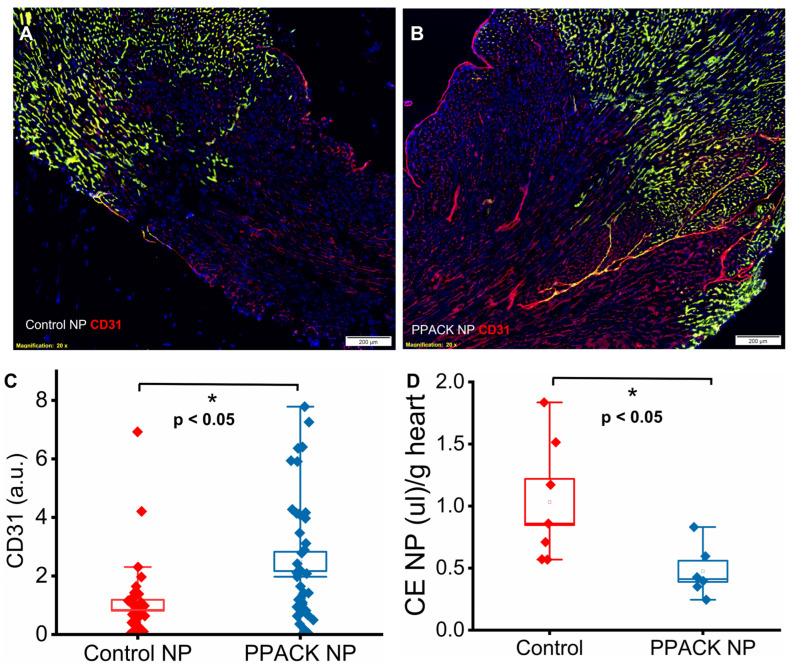
PPACK perfluorocarbon nanoparticles protected micro-vasculature. (**A**) Immunofluorescent image of CD31 staining (red) in control NP-treated mouse. (**B**) Immunofluorescent image of CD31 staining (red) of the PPACK NP treated mouse. The size of the scale bar was 200 µm, and the magnification was 20×. Green fluorescence signal indicates blood vessels in the normal region of the heat. DAPI was used for nuclei staining (blue). Yellowish indicated blood vessels in the normal region registered by both CD31 staining (red) and Lycopersicon Esculentum lectin DyLight 488 (green). (**C**) Quantification of CD31 in border zones of mice treated with either control NP or PPACK NP (*n* = 3 mice per group; *n* = 41 PPACK NP and *n* = 42 control NP regions of interest. Mean ± SEM; nonparametric analysis on *n* = 3). (**D**) Quantification of CE nanoparticles trapped in rat hearts after ischemia reperfusion injury. (*n* = 6 PPACK NP and *n* = 7 control NP treated whole hearts. Mean ± SEM; *: *p* < 0.05).

We hypothesized that the reduction in thrombin deposition with PPACK NP treatment prevented damage to the microvasculature that was a consequence of a strong local prothrombotic milieu. Accordingly, CD31 staining was performed to quantify vascular content in the border zone 24 h after ischemia reperfusion injury. In the normal perfused region, Lycopersicon Esculentum lectin DyLight 488 (green) and CD31 staining (red) colocalized to yield a yellow color (Figure 4A,B). In the border zones, CD31 signaling was significantly lower in mice receiving control NP (Figure 4A) versus PPACK NP treatments (Figure 4B). Normalized CD31 signals were 1 ± 0.19 vs. 2.50 ± 0.33 for Control NP vs. PPACK NP treated mice, respectively, indicating 2.5-fold greater vascular endothelial content after PPACK NP treatment (*p* < 0.05).

Protection of vasculature by PPACK NP was also observed in rat hearts by 2 h after ischemia-reperfusion injury using ^19^F MRS quantification in whole hearts ex vivo. Rats (*n* = 13) were treated with either saline plus CE NP or PPACK PFOB nanoparticles plus CE NP 15 min before ischemia induction and then euthanized 2 h after reperfusion. Hearts were excised and perfused with saline before ^19^F MRS quantification. The accumulation of CE NP in rats receiving saline or PPACK NP was 0.92 ± 0.19 µL/g vs. 0.47 ± 0.09 µL/g of heart tissue, respectively (*p* < 0.05). The results indicate that PPACK NP reduced the accumulation and trapping of CE NP by 49% as compared to saline treatment. As PFC NP only escapes from leaky or damaged vasculature [33,34,35], microvascular damage likely was substantially diminished.

### 2.5. PPACK PFC NP Treatment Reduced Border Zone Endothelial Activation

**Figure 5:**
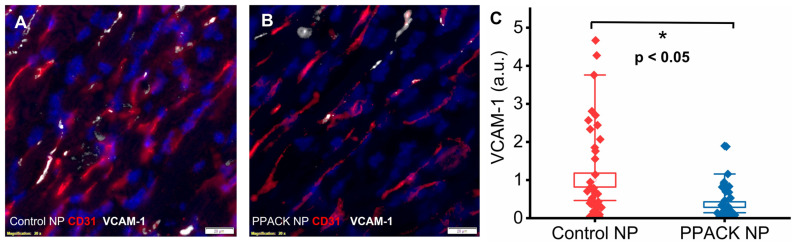
PPACK PFC NP treatment suppresses endothelial activation. (**A**) Immunofluorescent image of dual VCAM-1 (white) and CD31 (red) staining in heart section from control NP treated mouse. (**B**) Immunofluorescent image of VCAM-1 (white) and CD31 (red) staining in heart section from the PPACK NP treated mouse. DAPI was used for nuclei staining (blue). The size of the scale bar was 20 µm, and the magnification was 20×. (**C**) Quantification of VCAM-1 in the border zone regions of mice treated with either control NP or PPACK NP (*n* = 3 mice per group; *n* = 42 PPACK NP, and *n* = 44 control NP regions of interest. Mean ± SEM; nonparametric analysis on *n* = 3; *: *p* < 0.05).

To delineate the state of endothelial activation in border zone regions, double staining of CD31 and VCAM-1 was performed. Colocalization of CD31 and upregulated VCAM-1 was observed in border zones 24 h after ischemia reperfusion injury. Significantly more VCAM-1 was detected in control NP-treated hearts (Figure 5A) than in PPACK NP-treated hearts (Figure 5B). Normalized VCAM-1 signal was 1 ± 0.18 vs. 0.36 ± 0.07 (*p* < 0.05) in border zones of control NP vs. PPACK NP treated mice, respectively, which indicates that PPACK NP treatment suppressed VCAM-1 expression by 64%.

### 2.6. PPACK PFC NP Treatment Reduces Border Zone Inflammasome Signaling

**Figure 6:**
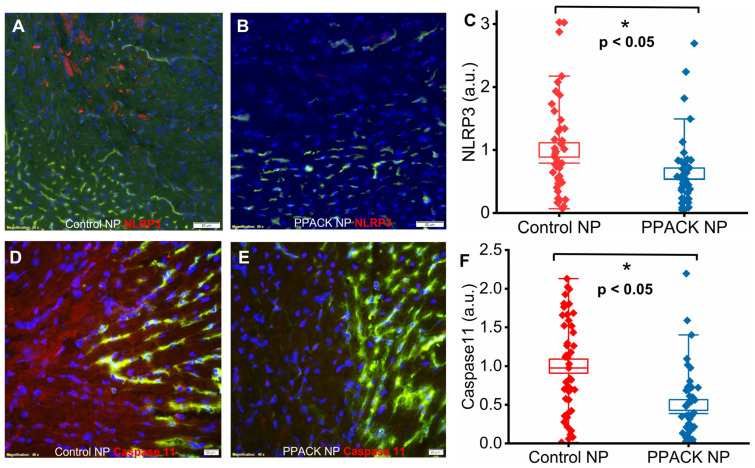
PPACK PFC NP treatment reduces inflammasome activation. (**A**) Immunofluorescent image of NLRP3 (red) staining in control NP-treated hearts. (**B**) Immunofluorescent image of NLRP3 (red) staining in PPACK NP treated hearts. Green fluorescence signal indicates blood vessels in the normal region of the heat. DAPI was used for nuclei staining (blue). The size of the scale bar was 50 µm, and the magnification was 20×. (**C**) Quantification of NLRP3 in border zones from control NP or PPACK NP treated hearts (*n* = 3 mice per group; *n* = 42 PPACK NP, and *n* = 45 control NP regions of interest. Mean ± SEM; nonparametric analysis on *n* = 3). (**D**) Immunofluorescent image of caspase 11 (red) staining in control NP-treated hearts. (**E**) Immunofluorescent image of caspase-11 (red) staining in PPACK NP treated hearts. Green fluorescence signal indicates blood vessels in the normal region of the heat. DAPI was used for nuclei staining (blue). The size of the scale bar was 20 µm, and the magnification was 40×. (**F**) Quantification of caspase-11 in border zones from control NP or PPACK NP treated hearts (*n* = 3 mice per group; *n* = 45 PPACK NP, and *n* = 45 control NP regions of interest. Mean ± SEM; nonparametric analysis on *n* = 3; *: *p* < 0.05).

Canonical and noncanonical inflammasome pathway activation was evaluated based on NLRP3 and murine caspase-11 expression. Significantly more NLRP3 and caspase-11 were detected in hearts from control NP-treated (Figure 6A,D) than from PPACK NP-treated mice (Figure 6B,E). Normalized NLRP3 signal was 1 ± 0.11 vs. 0.63 ± 0.09 in border zones from control NP vs. PPACK NP treated mice, respectively, which indicates that PPACK NP treatment reduced NLRP3 expression by 37% (*p* < 0.05). Normalized caspase-11 signal was 1 ± 0.09 vs. 0.50 ± 0.07 in the border zone for control NP vs. PPACK NP treated mice, respectively, which indicates that PPACK NP treatment reduced caspase-11 expression by 50% (*p* < 0.05).

### 2.7. PPACK PFC NP Treatment Reduced Border Zone Pro-Inflammatory Cytokines

**Figure 7:**
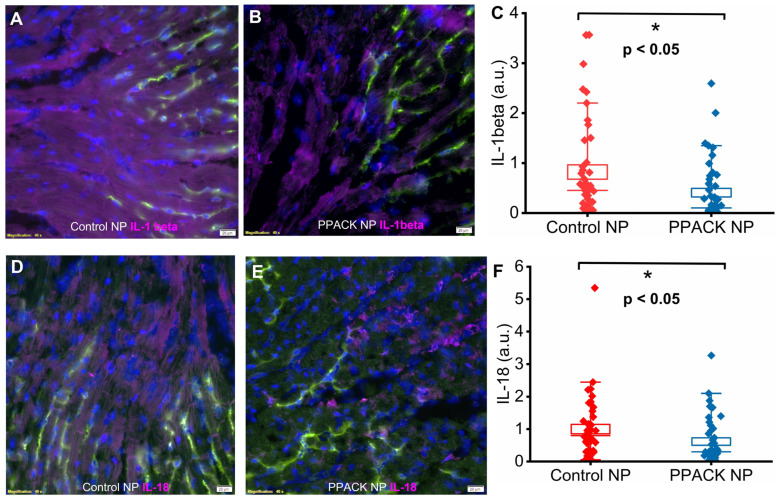
PPACK PFC NP treatment inhibits pro-inflammatory cytokine expression. (**A**) Immunofluorescent image of IL-1beta (purple) staining in control NP-treated hearts. (**B**) Immunofluorescent image of IL-1beta (purple) staining in PPACK NP treated hearts. Green fluorescence signal indicates blood vessels in the normal region of the heat. DAPI was used for nuclei staining (blue). The size of the scale bar was 20 µm, and the magnification was 20×. (**C**) Quantification of IL-1beta signal in border zones of control NP vs. PPACK NP hearts (*n* = 3 mice per group; *n* = 45 PPACK NP and *n* = 45 control NP regions of interest. Mean ± SEM; nonparametric analysis on *n* = 3). (**D**) Immunofluorescent image of IL-18 (purple) staining in control NP-treated hearts. (**E**) Immunofluorescent image of IL-18 (purple) staining in PPACK NP treated hearts. Green fluorescence signal indicates blood vessels in the normal region of the heat. DAPI was used for nuclei staining (blue). The size of the scale bar was 20 µm, and the magnification was 20×. (**F**) Quantification of IL-18 in border zones of control NP vs. PPACK NP hearts (*n* = 3 mice per group; *n* = 40 PPACK NP and *n* = 42 control NP regions of interest. Mean ± SEM; nonparametric analysis on *n* = 3; *: *p* < 0.05).

IL-1beta and IL-18 are potent pro-inflammatory cytokines triggered upon canonical and noncanonical activation of inflammasomes [36,37,38,39]. Significantly greater IL-1beta and IL-18 expression was observed in border zone regions from the mice receiving control NP treatment (Figure 7A,D) vs. PPACK NP treatment (Figure 7B,E). Normalized IL-1beta signal was 1 ± 0.24 vs. 0.45 ± 0.09 in border zones of control NP vs. PPACK NP treated mice, respectively, which indicates that PPACK NP reduced IL-1beta expression by 55% (*p* < 0.05). Normalized IL-18 signal was 1 ± 0.15 vs. 0.61 ± 0.12 in border zones of control NP vs. PPACK NP treated hearts, respectively, which indicates that PPACK NP treatment reduced IL-18 expression by 39% (*p* < 0.05).

## 3. Discussion

Perfluorocarbon (PFC) nanoparticles were originally developed and FDA-approved as a blood substitute in liter quantities [40,41] and for cardiac protection during angioplasty [42] based on the enhanced solubility of oxygen in the PFC core component [43]. More than 25 years ago, our laboratory adapted PFC NP as the first molecular imaging agent for ultrasound [44,45] and for paramagnetic (Gd^3+^) and fluorine (^19^F) MRI [46,47,48]. Subsequently, PFC NP was formulated as a theranostic antiinflammatory agent [49,50] and a highly potent but localized thrombin inhibitor [21,22,23,35,51,52].

When formulated as PPACK complexed antithrombin agents, we have reported critical safety data demonstrating no organ toxicity, immune cell induction, cytokine release, complement activation, or blood count abnormalities [35]. Bleeding times and clotting parameters all return to normal within 30–60 min after systemic PPACK NP delivery. Moreover, thrombin-bound particles may prevent further bleeding in damaged vasculature by coalescing to restore an antithrombotic surface on damaged microvasculature [35]. Although PPACK itself is an irreversible inhibitor of thrombin with sub-nanomolar affinity against thrombin, it is less active by orders of magnitude against other proteases, such as plasmin [21], which enhances its safety profile when delivered locally in nanoparticle format. A recent clinical research study identified hematological as well as coagulation parameters with predictive value in the prognosis of acute coronary syndromes [53]. Therefore, the preclinical safety profile of the PPACK PFC NP indicated that it could be a viable, safe candidate for further clinical translation.

In the present work, we evaluated the potential of PPACK PFC NP to preserve cardiac function and prevent ventricular remodeling after ischemia reperfusion injury. Our results confirm that highly potent PPACK NP treatment is indeed beneficial in this setting and operates in part by reducing inflammatory pathways that compromise vascular integrity. Vascular damage in acute myocardial infarction is characterized by marked sloughing of endothelial cells after coronary artery occlusion, creating an insult to vascular barrier integrity that exposes a hypercoagulable plaque milieu [13]. Exposed tissue factor (TF) activates thrombin that initiates platelet deposition, inflammation, and the production and activation of matrix metalloproteases that participate in ventricular remodeling [54]. Microvascular obstruction in the area at risk reportedly increases throughout the initial hours following reperfusion [55], suggesting that any treatment intended to protect vascular integrity should be delivered quickly upon reperfusion and should persist (with no washout) after reflow. Our results confirmed that PPACK NP could access the area-at-risk (Figure 1), where they maintain long-acting but local thrombin inhibition. Prevention of vascular pruning and preservation of vascular barrier function in the area at risk after the ischemia reperfusion injury (Figure 4) is likely an important factor contributing to the protection of viable but jeopardized cardiomyocytes and improved wound healing after reperfusion.

Inflammation-coagulation interactions may elicit prolonged regional ischemia/hypoxia that may progressively endanger jeopardized myocytes in the risk territory over some time interval after reperfusion. Thrombin induces inflammation and reactive oxidant species operating in part through inducible NOX pathways (NADPH oxidase) and is a key enhancer of endothelial permeability [56]. For example, thrombin acts through PAR-1 to destabilize endothelial cell tight junctions by downregulating claudin [57]. We propose that immediate mitigation of microvascular thrombosis with potent nanoparticle thrombin antagonists [21,22,23,35,58] can limit barrier damage at the endothelium by inhibiting thrombin-induced inflammatory cascades. It has been well documented that thrombin stimulates endothelial expression of adhesion molecules such as VCAM-1 [59,60,61], which are key early promoters of inflammatory leukocyte ingress. Our results (Figure 5) confirm that PPACK NP reduced vascular endothelial activation concomitant with the suppression of inflammatory signaling.

Thrombin also drives canonical NLRP3 inflammasome activation through its receptor, PAR4, in the heart [62]. It has been reported that in a mouse ischemia reperfusion injury model, NLRP3 inflammasome formation could be detected 24 h after reperfusion. Moreover, inhibition of NLRP3 inflammasomes is beneficial for limiting inflammation and infarct size [63]. Previous reporting has shown that NLRP3-deficient mice exhibit improved cardiac function after ischemia reperfusion injury [64]. In addition to the role of canonically activated NLRP3 inflammasomes, noncanonical caspase-11 inflammasomes also contribute to myocardial ischemia reperfusion injury via caspase-11/gasdermin signaling and damage related to pore formation in cellular membranes [65]. Activation of both NLRP3 inflammasome and caspase-11 inflammasome lead to the secretion of potent proinflammatory cytokines, IL-1beta and IL-18 [66], which are well recognized as potential therapeutic targets for acute myocardial infarction [67,68,69]. Our results, consistent with previous studies, demonstrated that PPACK PFC NP therapy inhibits canonical NLRP3 and noncanonical caspase-11 inflammasome pathways (Figure 6) and their downstream proinflammatory cytokines, IL-1beta and IL-18 (Figure 7).

The multifunctional PPACK PFC NP simultaneously inhibits thrombosis and inflammation, two intertwined and crucial factors in myocardial infarction, reperfusion injury, and cardiac remodeling. We contend that the responsible mechanism is related to the extraordinarily potent antithrombin effect of these locally acting nanoparticles. Our original reports of measured kinetic rates for thrombin inhibition by PPACK PFC NP indicated more than four orders of magnitude increased efficacy (2nd order rate constants) for nanoparticle conjugated PPACK versus free PPACK [21]. The major reason for this augmentation is the complexation of >10^4^ complexed PPACK on each nanoparticle when considered as a single antithrombin unit, as contrasted with a single free PPACK binding event that immediately exhausts any further antithrombin potential. Additionally, clots that form in the presence of PPACK NP appear more gelatinous, less contracted, and harbor far fewer platelets that are less activated and degranulated [21]. Finally, despite the extraordinary potency of PPACK PFC NP, the fact that a bleeding diathesis is established removes the necessity to design an antidote to counter bleeding risk, as is now the case for other novel anticoagulants. Moreover, the hydrophobic lipid layer of the PFC NP could stably carrier additional hydrophobic medications, such as a lipophilic statin. It has been reported that females could benefit more from statin if drug interactions could be addressed [70]. Since formulating statin into the lipid layer of PFC NP could prevent unwanted drug interactions, future studies could expand to multi-drug delivery by PFC NP.

There are certain limitations to this study. PPACK PFC NP was administered prior to induction of ischemia. It is probable that most of the blood containing any circulating nanoparticles will have drained from the occluded territory by the time of reperfusion, as these particles are vascularly constrained until vessel damage ensues upon reperfusion. For clinical use, we propose that the intravenous administration of this agent prior to angioplasty and coronary reperfusion may suffice to deliver sufficient concentrations of PPACK perfluorocarbon nanoparticles to areas at risk. On the other hand, our previous studies demonstrated that PPACK PFC NP treatment is beneficial in treating ischemia reperfusion injury-induced acute kidney injury administrated either before ischemia [22] or after the onset of the acute kidney injury [23], consistent with our argument above. It has been reported that cardiovascular risk is disproportionately affecting females more than males [70]. The evaluations from male mice were reported here and should be investigated in female mice in future preclinical studies.

## 4. Materials and Methods

### 4.1. Nanoparticle Formulation

The formulation of perfluorocarbon (PFC) nanoparticles was completed using the methods previously described [71] with modifications. Briefly, a lipid mixture of 98.7 mol% egg lecithin and 1.3 mol% dipalmitoyl-phosphatidylethanolamine (Avanti Polar Lipids, Piscataway, NJ, USA) was dissolved in the mixture of methanol and chloroform (1:3, *v*/*v*). For the fluorescent PFC nanoparticle, the lipid film was generated with 98.7 mol% egg lecithin, 1 mol% dipalmitoyl-phosphatidylethanolamine, and 0.3 mol% rhodamine (1,2-dioleoyl-sn-glycero-3-phosphoethanolamine-N-(lissamine rhodamine B sulphonyl) (Avanti Polar Lipids, Piscataway, NJ, USA). For the PPACK precursor PFC nanoparticles, the lipid film included 98.5 mole% egg lecithin and 1.5 mole% 1,2-distearoyl-sn-glycero-3-phosphoethanolamine-N-[carboxy(polyethylene glycol)-2000] (Avanti Polar Lipids, Piscataway, NJ, USA). The solvents were removed under reduced pressure to generate a lipid film with rapamycin, which was dried in a vacuum oven overnight. Next day, the lipid film (2.0%, *w*/*v*), either perfluorooctyl bromide (PFOB) or Perfluoro 15-Crown-5 Ether (CE) (Gateway Specialty Chemicals, St. Peters, MO, USA) (20%, *w*/*v*), and MilliQ water were sonified and emulsified at 20,000 psi for 6 passes in an ice bath (LV-1 Microfluidics emulsifier; Microfluidics, Newton, MA, USA). For therapeutic applications, PFOB was used as the hydrophobic core of the perfluorocarbon nanoparticles. For ^19^F magnetic resonance spectroscopy and magnetic resonance imaging, CE served as the hydrophobic core.

### 4.2. Formulation of PPACK PFC Nanoparticles

Amine-carboxyl coupling was utilized for functionalizing PFC nanoparticles containing 1,2-distearoyl-sn-glycero-3-phosphoethanolamine-N-[carboxy(polyethylene glycol)-2000] with PPACK. After one hour of mixing 2 mL PFC nanoparticles with 25 mg PPACK, EDCI 1-[3-(Dimethylamino)propyl]-3-ethylcarbodiimide methiodide (4 mg) was added for overnight coupling. Excess PPACK and EDCI were removed by dialysis (MWCO 3000–5000). The extent of PPACK coupling was determined by reverse-phase HPLC quantification of uncoupled PPACK after centrifugation of nanoparticles with Cleanascite lipid adsorption reagent (Agilent Technologies, Santa Clara, CA, USA). Elution of PPACK in a C18 column was achieved with an isocratic method employing 9.9% acetonitrile, 0.089% trifluoroacetic acid, and 90.011% water. PPACK was detected via phenylalanine absorbance (258 nm).

### 4.3. Nanoparticle Distribution and Zeta Potential Measurement

The size distributions of the PFC nanoparticles were evaluated by dynamic light scattering (Brookhaven Instruments Corp., Holtsville, NY, USA). The surface charges of the nanoparticles, indicated by zeta-Potential values, were determined with a PALS Zeta Potential Analyzer (Brookhaven Instruments Corp.). Data were collected in the mode of phase-analysis light-scattering (PALS) after the solution was equilibrated at 25 °C. All samples were diluted in MilliQ water (Merck, Rachway, NJ, USA).

### 4.4. Acute Myocardial Infarction Animal Model

#### 4.4.1. Mouse Acute Myocardial Infarction Model

**Figure 8:**
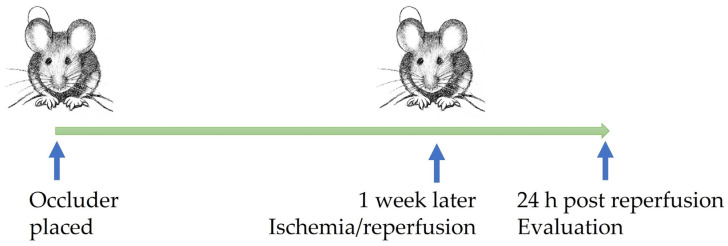
Scheme of mouse AMI procedure timeline.

A closed-chest mouse model of cardiac ischemia reperfusion injury was generated, as published previously [72]. Briefly, 12 weeks old male C57BL/6Jmice (Jackson Laboratory, Bar Harbor, ME, USA) were anesthetized with ketamine/xylazine −100/10 mg/kg, intubated, and mechanically ventilated. A midline incision was made, exposing the heart, and an 8–0 Prolene suture was then placed around the proximal LAD to maximize the ischemic area. The suture was then threaded through a 1 mm piece of polyethylene tubing, forming a loose snare that served as the occluder. Each end of the suture was exteriorized through the thorax and stored in a subcutaneous pocket. The skin was then closed over the exteriorized suture ends with 6–0 Prolene. Instrumented mice were allowed to recover for 1 week before induction of ischemia (Figure 8). A baseline echocardiogram was then performed that ensured that LAD ligation had not occurred. Ischemia was induced after anesthetizing the animals with 1.5% isoflurane, opening the skin over the subcutaneous pocket, and exposing the exteriorized suture. The suture ends were dissected away from any subcutaneous tissue, and tension was exerted until ECG ST-segment elevation was observed. Ischemia was confirmed by visualizing a new wall motion abnormality using simultaneous echocardiography. Following 90 min of ischemia time, tension was released, and the suture ends were placed back into the subcutaneous pocket. The skin was then closed, as described earlier. All the animal-related procedures were approved by the Washington University School of Medicine IACUC and conducted by the Mouse Cardiovascular Phenotyping Core.

#### 4.4.2. Rat Acute Myocardial Infarction Model

An open chest rat model of cardiac ischemia reperfusion injury was generated, as published previously [73]. Briefly, 12 weeks old male Sprague–Dawley retired breeder rats were purchased from Harlan Laboratories Inc. (Indianapolis, IN, USA). The rats were anesthetized by intraperitoneal injection of 40 mg/kg pentobarbital sodium, which was supplemented as needed to maintain a deep surgical plane of anesthesia. Throughout the procedures, the rat’s body temperature was maintained at 37 °C with ECG monitoring. The rats were ventilated with room air, and a tracheostomy was performed. In addition, the tail vein was cannulated. An intercostal thoracotomy was performed to expose the basal region of the heart, and the left coronary artery was ensnared with a 5–0 polypropylene suture by taking an intramyocardial stitch at the distal edge of the left atrial appendage. Additional silk sutures were tied to each arm of the polypropylene suture to facilitate the release of the occlusion knot. All the animal-related procedures were approved by the University of South Florida IACUC.

### 4.5. Tissue Collection and Preservation

Mouse hearts were excised with all vessels tied and perfused with PBS through a gavage needle secured to the aorta. After the PBS (Fisher Scientific, Waltham, MA, USA) perfusion, the occluder was tied again before the perfusion with 1:6 diluted Lycopersicon Esculentum lectin with DyLight 488 (Vector Laboratories, Newark, CA, USA) in PBS (Fisher Scientific, Waltham, MA, USA). The heart slices were preserved in O.C.T. (Fisher Scientific, Waltham, MA, USA) and stored at −80 °C before cryosection. Rats were systematically perfused with saline before the hearts were collected and stored in PBS on ice before MRI and MRS evaluation.

### 4.6. Echocardiogram

A mouse echocardiogram was performed at Washington University by the Mouse Cardiovascular Phenotyping Core facility using the VisualSonics 2100 Echocardiography System (VisualSonics Inc., Toronto, ON, Canada). Avertin (0.005 mL/g) was used for sedation based on previously established methods [74]. Both 2-dimensional and M-mode images were obtained in the long- and short-axis views. LV dimensions were calculated using standard techniques and were normalized to body weight. Measurements were performed on 3 independently acquired images per animal by investigators who were blinded to the experimental group.

### 4.7. ^19^F Magnetic Resonance Spectroscopy and Magnetic Resonance Imaging

MR images were acquired on a 7T horizontal magnet (Agilent ASR 310) and (Bruker Biospin, Inc. BioSpec AV3HD, Billerica, MA, USA), using a homemade Transmit-receive coil 12 mm diameter. The same coil was tuned to ^1^H and ^19^F for each image acquisition using an external tuning box. The tuning was carefully accomplished without moving the coil or sample to facilitate ^1^H-^19^F image co-registration.

Anatomical T2-weighted images were acquired with a TurboRARE sequence (RARE factor = 16) with repetition time/echo time (TR/TE) = 3900/36 ms, a field of view (FOV) = 20 × 20 mm^2^, image size 128 × 128, slice thickness of 0.5 mm, and 25 slices to cover the entire heart in long axis. T2* maps were acquired with the same image resolution with a multiple gradient echo sequence (TR = 3476, 48 echoes spaced by 2.81 ms (TE = 2.6 to 134.67 ms)). In addition, 19F spin density images were acquired with the RARE sequence (RARE factor 8) with TR/TE = 2000/4.51 ms, 2056 averages, and the same image resolution as all other sequences for image co-registration.

### 4.8. Histology

Immunofluorescence staining was performed on O.C.T-embedded frozen kidneys, which were sectioned at 8 µm before staining. Kidney sections were fixed in 4% PFA (Fisher Scientific, Waltham, MA, USA) before staining. Primary antibodies of thrombin, CD31, VCAM-1, NLRP3, Caspase-11, IL-1beta, or IL-18 (Abcam, Waltham, Boston, MA, USA) were applied onto the frozen section for 1 h at room temperature, followed by the corresponding secondary antibodies against rabbit labeled with Alex 594 or Cy5.5 (Abcam, Waltham, Boston, USA) incubation at room temperature for 30 min. Slides were then mounted with VECTASHIELD antifade mounting medium with DAPI (Vector Laboratories, Newark, CA, USA) before being imaged with an Olympus dark-field microscope outfitted with a HAMAMATSU digital camera C11440 at 20× or 40× magnification. Double-blind data acquisition was performed on all images.

### 4.9. Statistics

Results are expressed as mean ± standard error of the mean (SEM). *T*-test and nonparametric analysis were used. Statistical significance of differences was attributed at *p* < 0.05.

## Data Availability

Not applicable.
